# Immunogenicity of SARS-CoV-2 mRNA Vaccine in Breast Cancer Patients Undergoing Active Treatment: A Prospective Observational Study

**DOI:** 10.3390/pathogens14090947

**Published:** 2025-09-18

**Authors:** María Leonor Fernández-Murga, Lucía Serrano-García, Giuseppe D’Auria, María Portero Hernández, Llúcia Martínez-Priego, Loreto Ferrús-Abad, Griselda de Marco, María Victoria Domínguez-Márquez, Antonio Llombart-Cussac

**Affiliations:** 1 Molecular Oncology Laboratory, Arnau de Vilanova Hospital, Foundation for the Promotion of Health and Biomedical Research of the Valencian Community (FISABIO), 46015 Valencia, Spain; luchiserga@gmail.com (L.S.-G.); mariaportero@gmail.com (M.P.H.); antonio.llombart@maj3.health (A.L.-C.); 2 Sequencing and Bioinformatics Service, Foundation for the Promotion of Health and Biomedical Research of the Valencian Community (FISABIO), Avda. de Catalunya 21, 46020 Valencia, Spain; Giuseppe.Dauria@fisabio.es (G.D.); llucia.Martinez@fisabio.es (L.M.-P.); loreto.Ferrus@fisabio.es (L.F.-A.); GriseldadeMarco@fisabio.es (G.d.M.); 3 Microbiology Department, Arnau de Vilanova Hospital, Foundation for the Promotion of Health and Biomedical Research of the Valencian Community (FISABIO), 46015 Valencia, Spain; m.victoria.dominguez@uv.es

**Keywords:** SARS-CoV-2, mRNA vaccine, antibodies, breast cancer, microbiome

## Abstract

Understanding the immune response to SARS-CoV-2 vaccination in cancer patients remains a critical priority given their immunocompromised status. In this prospective observational study, we evaluated humoral and cellular immunity across three time points—baseline, post-second dose, and post-booster—in 23 breast cancer patients undergoing active treatment. IgG antibody levels showed a significant increase following vaccination, with a 300-fold rise after the second dose and a 2200-fold increase post-booster, indicating a strong humoral response. CD19^+^ B cells also increased significantly, supporting B cell-mediated activation. Although overall T cell frequencies remained stable, we observed a shift toward memory phenotypes, with decreased naïve CD4^+^ and CD8^+^ T cells and increased central and peripheral memory subsets after the booster. Notably, CD8^+^ TEMRA cells expanded significantly, suggesting cytotoxic memory formation. Correlation analyses linked peripheral memory CD4^+^ T cells with anti-SARS-CoV-2 IgG titers, while CD8^+^ TEMRA cells showed an inverse association. Antigen-specific CD8^+^ T cell response was evaluated using APC-labeled MHC I Dextramer reagents. After the booster, 55.5% of patients developed detectable antigen-specific CD8^+^ T cells, whereas 44.5% did not. Importantly, one patient who failed to develop antigen-specific CD8^+^ T cells experienced a mild SARS-CoV-2 infection, suggesting that the absence of this response may increase susceptibility despite high IgG levels. These findings indicate that antigen-specific CD8^+^ T cell responses and antibody levels may act as complementary but not directly correlated arms of immunity. Microbiota profiling via sPLS-DA suggested weak but distinct microbial signatures associated with immune responsiveness, particularly enrichment of taxa such as *Alistipes* and *Romoutsia* among high-antibody responders. These findings emphasize that SARS-CoV-2 vaccination is immunogenic and well tolerated in breast cancer patients under therapy and highlight the need to further explore microbiota–immune interactions to optimize vaccination strategies in oncology.

## 1. Introduction

Cancer patients are particularly vulnerable to infectious diseases, not only due to the immunosuppressive effects of the malignancy itself but also as a result of cytotoxic antineoplastic treatments. Among them, individuals with breast cancer receiving chemotherapy, immunotherapy, or targeted agents may exhibit impaired immune function, which raises concerns about their ability to mount effective responses to vaccines—especially in the context of the ongoing COVID-19 pandemic. While SARS-CoV-2 vaccines have been shown to be generally safe and capable of inducing immune responses in cancer patients, the magnitude and durability of such responses vary depending on cancer type, treatment regimens, and patient-specific factors [1,2,3,4].

In healthy individuals, mRNA-based vaccines elicit robust humoral and cellular immunity through activation of B cells, CD4^+^ helper T cells, and CD8^+^ cytotoxic T cells [5,6,7]. However, in oncologic populations, these immune responses may be diminished due to treatment-induced immunosuppression, resulting in lower antibody production and weaker T cell responses. Although early studies in hematologic and solid tumors primarily focused on humoral immunity, there is growing interest in characterizing T cell–mediated responses and long-term immunological memory in cancer patients after vaccination [8,9,10]. Understanding the dynamics of cellular immunity is essential to inform booster strategies and adapt vaccine protocols for immunocompromised individuals.

Beyond the immune system itself, recent evidence has highlighted the gut microbiota as a key player in vaccine responsiveness. Specific microbial taxa—particularly those that produce short-chain fatty acids (SCFAs)—have been implicated in modulating antigen presentation, dendritic cell activation, and the differentiation of follicular helper T cells (Tfh), which are all crucial for effective immune responses to vaccination [11,12,13]. In cancer patients, the gut microbiome is frequently altered by chemotherapy, antibiotics, and dietary changes, which may influence both vaccine immunogenicity and broader clinical outcomes [14,15,16].

Despite their clinical relevance, integrated analyses assessing humoral and cellular responses alongside microbiota composition in patients with breast cancer remain limited. Few prospective studies have examined immune dynamics following multiple vaccine doses in this specific population undergoing active treatment [17,18]. This gap is especially important given that emerging data suggest that, although breast cancer patients may have delayed or reduced immune responses, they still derive substantial benefit from vaccination in terms of reduced severity and hospitalization risk [19,20].

In this prospective observational study, we aimed to characterize the immunogenicity of SARS-CoV-2 mRNA vaccines in breast cancer patients by measuring antibody titers, lymphocyte subpopulations, and memory T-cell phenotypes longitudinally, after primary and booster immunizations. Additionally, we explored the association between vaccine-induced immune responses and gut microbiota composition using multivariate statistical approaches.

Understanding how cancer therapy and microbiota dysbiosis influence vaccine efficacy is essential to improve protection strategies for this vulnerable population. While COVID-19 vaccines activate both arms of the immune system, these effects may be modulated by host microbiota diversity and the immunological state induced by treatment. Maintaining or restoring a balanced gut microbiome—possibly through probiotics, prebiotics, or dietary interventions—might enhance vaccine responses, although further research is needed to confirm these approaches. As we continue to navigate the pandemic, integrating immunological and microbiome insights will be critical for developing more effective, personalized vaccination strategies for patients with breast cancer and other malignancies [21].

## 2. Materials and Methods

### 2.1. Study Design and Participants

**Figure 1:**
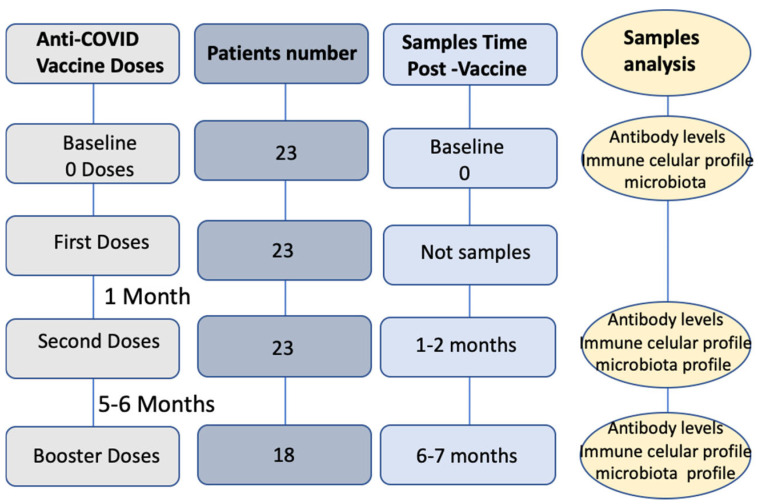
Study design: Collection times of blood and feces samples for determination of cellular and humoral immune response and microbial composition, respectively.

This study was carried out at a single institution, Hospital Arnau de Vilanova de Valencia of Spain (HAV). In this prospective follow-up study (conducted between May 2021 and April 2022), we recruited 23 patients with documented advanced breast cancer undergoing treatment at the Department of Medical Oncology of the HAV. For enrollment, patients had to have received two doses, as well as a booster dose, of the approved anti-SARS-CoV-2 mRNA vaccines BNT162b2 or mRNA5-1273. Study subjects had to meet a series of criteria: patients were excluded if they had any other comorbidity known to be associated with immunosuppression (e.g., human immunodeficiency virus infection), if they received immunosuppressive treatment for a reason other than neoplastic disease, or if they had received another vaccine in the preceding six months. Patients with any known previous COVID-19 infection, either symptomatic or asymptomatic and documented by positive (>100 BAU/mL) anti-nucleocapsid titers, were also excluded from participation. Clinical data, demographic characteristics, and a post-vaccination symptom questionnaire (symptoms: cough, fever, dyspnea, sore throat, vomiting, diarrhea, headache, asthenia, anosmia, severity and duration of symptoms, need for hospital admission, need for intensive care, treatment received, and outcome (recovery or death)) were collected. Blood samples were obtained just prior to the administration of the vaccines; SARS-CoV-2 IgG (Anti-Spiga) antibody and lymphocyte subpopulations and their differentiation stages were analyzed at different times (see Figure 1). A study of the gut microbiota by means of full-length 16S rDNA gene amplification and sequencing using Pacific Biosciences (Sequel II System) was also carried out on fecal samples from different phases of the study (see below). Blood and stool were collected from in-hospital patients by hospital staff. Stool was collected in sterile collection tubes and stored at −80 °C until processing.

### 2.2. Ethical Considerations

This study was conducted in accordance with the declaration of Helsinki. The Ethics Committee of Hospital Arnau de Vilanova de Valencia approved this study (Ethics Committee No. HAV-BAR-2020-03). At all times, the current legislation on data confidentiality was followed, namely the Organic Law 03/2018 on Data Protection of 5 December 2018, published in BOE no. 294, BOE-A-2018-16673.

The anonymity of the subjects participating in this study was ensured. The authors have no relevant affiliations with any organization or entity with a financial interest in the subject matter or materials discussed in the manuscript. All patients enrolled in this transversal study provided informed consent. 

### 2.3. Blood Sample Analysis

#### 2.3.1. Flow Cytometry Immunophenotyping

Blood cell counts were obtained using an LH750 cell counter (Beckman Coulter, Inc., Fullerton, CA, USA). Mononuclear cells (MNCs) were enriched via density gradient centrifugation of an EDTA-anticoagulated blood sample with Lymphoprep™ (Palex Mediacal SA, Sant Cugat del Vallès, Spain), spun at 3500 rpm for 20 min. After two washes in Phosphate-Buffered Saline (PBS), the cells were resuspended in 200 mL of PBS. In order to evaluate the lymphocyte populations, the MNCs were incubated with the following monoclonal antibodies conjugated with fluorochromes (Beckman Coulter, Inc., Miami, FL, USA): CD19-PE (clon: J3-119), CD45RA-ECD (clon: 2H4LDH11LDB9 (2H4)), CD56-PC7 (clon N901 (NKH-1)), CD62L-APC (clon: DREG56), CD4-APC A750 (clon 13B8.2), CD3-APC A700 (clon UCHT1), CD8-PB (clon B9.11), and CD45-KRO (clon J33). 

Following a 10-min incubation at room temperature, 1 mL of freshly prepared VersaLyse “Fix-and-Lyse” reagent (Beckman Coulter, Inc.) was added. The suspension was mixed and incubated and then protected from light for 20 min at room temperature. After the addition of 2 mL PBS and centrifugation at 1500 rpm for 5 min (room temperature), the supernatant was aspirated and the cell pellet resuspended in 0.5 mL PBS. Acquisition and analysis were performed on a Navios flow cytometer (Beckman Coulter, Inc.) using Kaluza Software (version 2.2.1), with 100,000 events recorded per sample. Absolute counts of circulating cell subsets were calculated using a dual-platform counting technology. 

#### 2.3.2. Detection of IgG Antibodies Against SARS-CoV-2

Detection of IgG antibodies against SARS-CoV-2 was performed using the SARS-CoV-2 IgM and SARS-CoV-2 IgG II Quant assays, with the corresponding calibrators and controls for the ARCHITECT i2000SR analyzer (Abbott Diagnostics, Lake Forest, IL, USA). The ARCHITECT System uses chemiluminescent microparticle immunoassay (CMIA) technology as a detection method to measure and quantify the concentration of antibody present in the serum. The SARS-CoV-2 IgG II Quant assay quantifies IgG in BAU/mL and provides standardized values in accordance with the WHO standard. We correlated the IgG values with the WHO international standard 20/136, thus obtaining the units in BAU/mL (BAU: Binding Antibody Units). The cutoff level of a positive IgG result was ≥7.1 BAU/mL. 

#### 2.3.3. Detection of Specific SARS-CoV-2 CD8^+^ T Cell Response 

To identify antigen-specific CD8^+^ T cells, the SARS-CoV-2 spike protein-specific reagent MHC I Dextramer®, HLA-A*0201 (Immudex®, DK), was used. The reagent contains a recombinant MHC class I complex loaded with an immunodominant peptide derived from the SARS-CoV-2 Spike protein (pMHC-I), restricted to the defined HLA allele A*0201 and the epitope YLQPRTFLL. These pMHC-I complexes are presented in multimeric form (Dextramer®), which increases the binding avidity and intensity of the fluorescent signal. When incubated with mononuclear cells (MNCs), only CD8^+^ T cells expressing a TCR specific for that Spike-HLA peptide will bind to the reagent. This binding is detected via flow cytometry, allowing for the identification, quantification, and characterization of the viral antigen-specific CD8^+^ T cell population.

The protocol was carried out according to the manufacturer’s instructions, and it has been validated in research publications [22,23]. Isolated MNCs (see Section 2.3.1) were washed twice and stained with 1X PBS supplemented with 5% fetal bovine serum (FBS). Half of the sample obtained was used for labeling with the negative control reagent Dextramer®. Staining with the Dextramer labeled with the fluorochrome APC was performed in the dark at room temperature (R/T) for 10 min and subsequently incubated for 20 min R/T in the dark with anti-CD8-FITC and anti-CD3-PE antibodies (Beckman Coulter, Inc). The samples were washed twice with 1X PBS + 5% FBS and lysed with 1 mL of Versalyse Lysing Solution (Beckman Coulter, Inc.). Then, after two washes with PBS, the cells were resuspended in 400 uL of PBS 1X and the entire volume was acquired in a Gallios 3L, 10C flow cytometer (Beckman Coulter, Inc., USA). Analysis was carried out using Kaluza Software (Beckman Coulter, Inc., USA). A percentage of Dextramer MHC-I CD8^+^ T cells ≥ 0.01 was considered positive. 

### 2.4. Microbiome Library Preparation and Sequencing

#### 2.4.1. DNA Extraction

A QIAamp PowerFecal Pro DNA Kit (Qiagen Ref.: 51804) was used for DNA extraction. The starting fecal amount was 250 mg. The final elution was performed in 75 uL of kit buffer. To start the preparation of the sequencing libraries, the extracted DNA samples were diluted to 0.4 ng/uL.

#### 2.4.2. Microbiome Library Preparation and Sequencing

The 27F (5′-AGRGTTYGATYMTGGCTCAG-3′) and 1492R (5′-RGYTACCTTGTTACGACTT-3′) universal primer set was used to amplify the full-length 16S rRNA gene from the genomic DNA [24,25]. Both the forward and reverse 16S primers were tailed with sample-specific PacBio barcode sequences to allow for multiplexed sequencing. The KAPA HiFi Hot Start DNA Polymerase (KAPA Biosystems, Boston, MA, USA) was used to perform 27 cycles of PCR amplification, with denaturing at 95 °C for 30 s, annealing at 57 °C for 30 s, and extension at 72 °C for 60 s. The amplification products were checked using the Fragment Analyzer (Agilent Technologies, Santa Clara, CA, USA) and then pooled at an equimolar concentration. The pool was processed with the SMRTbell Express Template Prep Kit 2.0 (PacBio, Menlo Park, CA, USA) following the manufacturer’s instructions for Multiplexed SMRTbell® Library Preparation and Sequencing (Part Number 101-599-700 Version 04, PACBIO). Before sequencing, primer annealing and polymerase binding steps were carried out using the Sequel II Binding Kit 2.1 and the Sequel II DNA Internal Control Complex 1.0 (PacBio, USA). Finally, sequencing was performed using the Sequel II Sequencing Kit 2.0 (PacBio, USA) on the Sequel II PacBio system.

### 2.5. Statistical Analysis 

#### 2.5.1. Blood Sample Analysis

Firstly, the data were coded and normality tests were performed for quantitative variables. Subsequently, a descriptive analysis was carried out; absolute and relative frequencies (percentages) were calculated for categorical variables, while median and interquartile ranges were calculated for quantitative variables. To explore the relationships and significant differences between variables, non-parametric tests, appropriate for the nature of the data, were implemented. Spearman’s correlation was used to assess the relationship between quantitative variables. The Mann–Whitney U test was used to compare immunity levels and other parameters between patients receiving chemotherapy and those receiving targeted therapy. The Friedman test was applied to assess differences in parameters measured at the three different time points (baseline, second, and booster doses). In addition, the Wilcoxon test was used for pairwise comparisons between study variables. To analyze the influence of the independent variables on immunity levels, a multiple linear regression model was fitted. The initial model included the independent variables of age, comorbidities, type of therapy, and tumor type. Using the backward elimination method, non-significant variables were progressively eliminated in an iterative process, until a final model with relevant variables was obtained. The Durbin–Watson statistic was used to ensure the independence of the residuals, and the adjusted coefficient of determination (adjusted R^2^) was used to assess the quality of the fit. In addition, advanced visualizations were generated for an analysis to complement the statistical analyses. A confidence level of 95% was established for all analyses, which were carried out using IBM SPSS Statistics version 25. Graphs were created and statistical analyses were performed using GraphPad Prism software version 6.0 for Windows (GraphPad Software, San Diego, CA, USA). 

#### 2.5.2. Microbiome Analysis

After PacBio 16S rDNA amplicon sequencing, denoising, filtering and trimming, and chimera depletion were performed using the DADA2 pipeline [26]. Taxonomic affiliations were assigned using the BLAST algorithm integrated into the quiime2 plugins against Silva138 [27]. Operative Taxonomic Units (OTUs), in terms of amplicon sequence variants from DADA2 pipeline results, and taxonomic data were organized in a *phyloseq* object [26]. Alpha diversity in terms of the observed taxa, the Chao1 estimator, and Shannon diversity indexes was calculated using *phyloseq* functions. OTU data at the species taxonomic level were normalized using central log transformation, adding a pseudocount of 1 using the mixOmics packages. Differential analysis between the groups was carried out using the sparse Partial Least Squares Discriminant Analysis (sPLS-DA) included in the mixOmics package [28,29]. The integration between microbiome data and humoral marker assays was performed using the *Diablo* integrative approach [30]. Microbiome analyses were carried out using the R-statistics environment (version 4.1.2; R Core Team, 2024).

## 3. Results

### 3.1. Patient and Disease Characteristics

A total of 23 patients, comprising those who received a second vaccine dose, were initially recruited and included in the analysis. However, the post-booster analysis was conducted on 18 patients, as 3 of the 23 patients declined the booster vaccination and 2 others withdrew from the study due to disease progression. 

The predominant vaccine administered, as both the second dose (60.9%) and the booster dose (66.7%), was mRNA-1273. BNT172b2 was administered to 39.1% of patients as the second dose and 33.3% as the booster dose.

Baseline patient and disease characteristics are summarized in Table 1. The median age at the time of the first vaccination was 54 years (range 45–76), and 48% of patients (*n* = 11) had luminal-subtype breast cancer. The majority of patients (48%, *n* = 11) had stable disease. All participants had advanced disease and were undergoing active treatment, as per the study’s inclusion criteria.

Regarding antineoplastic treatment, 48% of patients received targeted therapy (including anti-hormone therapy, immunotherapy, aromatase inhibitors, and tyrosine kinase inhibitors), while 52% were treated with chemotherapy. One patient (4%) died due to neoplastic disease. Comorbidities were generally uncommon. Additionally, the dataset consisted primarily of non-smokers (87%), and more than half were postmenopausal individuals (52%).

The study also included a microbiome analysis of the patients at three time points: baseline, post-second dose, and post-booster dose. Overall main alpha diversity indexes such as the Chao1 estimator and Shannon diversity did not highlight significant differences between subjects receiving chemotherapy and targeted therapy (Supplementary Figure S1). Grouping depending on treatment or by antibody response, independent of dose, did not have any influence on alpha diversity. 

### 3.2. Humoral and Cellular Response After Vaccine Doses

Table 2 summarizes the data on the patients’ immune parameters across three time points: baseline, after the second vaccine dose, and after the booster dose. Statistical comparisons using the Friedman test were used to evaluate changes over time, with corresponding p-values indicating the significance of the differences observed. 

We observed a marked increase in IgG levels after vaccination, peaking after the booster dose (2200 times, *p* = 0.0001), indicating a robust humoral immune response.

No significant changes were observed in CD3^+^CD4^+^ and CD3^+^CD8^+^ T cell and NK cell percentages across time points. However, we observed that the percentages of NKT cells changed significantly, with a 1.5-fold increase after the second dose and a 6.9-fold reduction after the booster (*p* < 0.0001). Consistent with the increase in IgG-anti COVID-19, a significant increase in CD19^+^ B cell percentages was observed (2-fold after the second dose and 2.85-fold after the booster dose, *p* = 0.033), reflecting B cell activation following vaccination.

The dynamics of CD3^+^CD4^+^ T cell differentiation stages during the vaccination period showed a significant reduction in naïve CD3^+^CD4^+^ T cells after boosting (*p* = 0.020), indicating T cell differentiation, and a significant increase (*p* = 0.020) in CD3^+^CD4^+^ T cells with central memory, reflecting a strong development of immune memory. As for the differentiation stages of CD3^+^CD8^+^ T cells, a significant reduction in naïve CD3^+^CD8^+^ T cells after boosting (*p* = 0.038) was also observed during the vaccination period, indicating a differentiation of T cells. However, we observed a significant increase in peripheral memory CD3^+^CD8^+^ T cells (*p* = 0.0001), highlighting an enhanced long-term immune response.

Significant increases in IgG levels, CD19^+^ B cells, and the central/peripheral memory T cell subsets indicate a strong adaptive immune response. In addition, naïve T cells decreased while memory T cells increased, reflecting effective immune activation and differentiation after vaccination.

Post-vaccination symptoms were mild, the most frequent being fever (21.7–20%), asthenia (47.8–26.7%), body aches (47.8–26.7%), pain at the puncture site (87–92.9%), and a transient reduction in usual activity (26.1–20%). In all cases, the medication consumed was antipyretic, and hospitalization was not required (Supplementary Table S1). 

**Figure 2:**
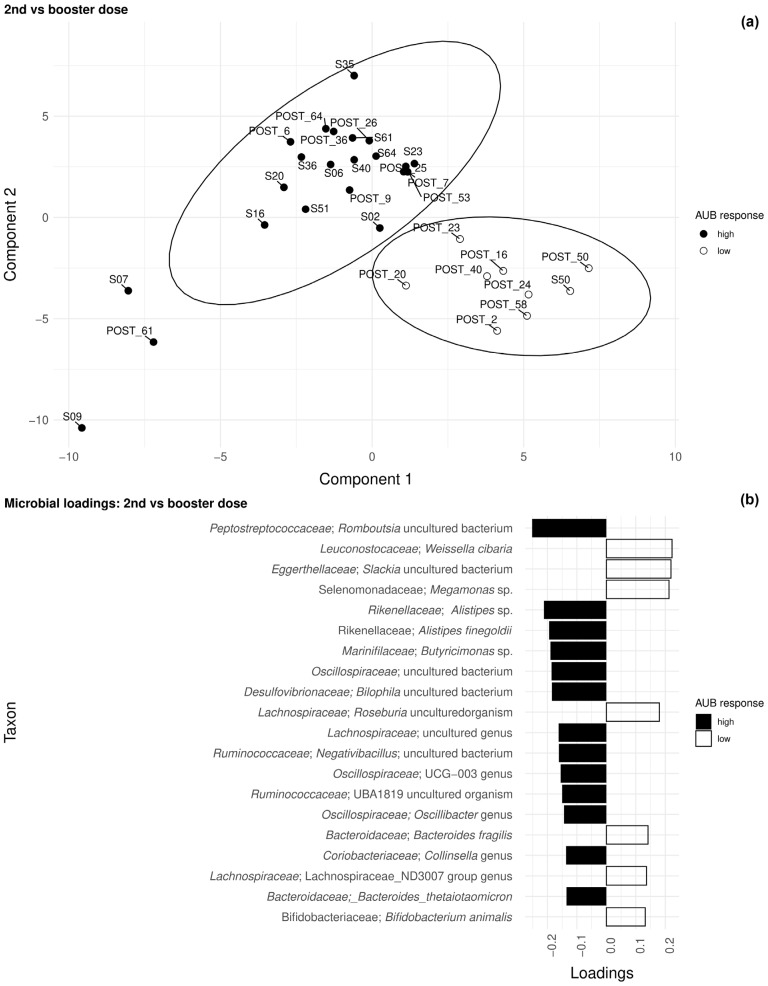
The patterns represent high and low responders after sPLS-DA model application. (**a**) Distances of the second dose versus booster samples plotted after the application of the sPLS-DA model. The plot includes 95% confidence ellipses. The samples were plotted using the first two components of the obtained model. (**b**) Importance (loadings) of main taxonomic groups ordered by relevance (top-down).

Regarding the microbiome analysis, the use of sPLS-DA allowed us to separate the samples by high and low antibody response at baseline compared with the second and booster doses (Figure 2). sPLS-DA reduction allowed us, albeit with very weak loading values below 0.2, to identify several taxa which may be relevant to discriminating between the three time points. For instance, the application of a more restrictive analysis based on the mixOmics fine-tuning protocol eliminated most of the taxa from the first comparison (baseline vs. second dose), except for a tag on one uncultured *Ruminococcus*. In the second comparison (second dose vs. booster dose), we found uncultured Romoutsia, Alistipes, and Butyricimans among the high responders and *Weissella cibaria*, *Slackia* sp., *Megamonas* sp., and *Roseburia* sp. among the low responders. Other taxa were found but had very low or non-significant differential abundance scores (Figure 2).

### 3.3. Humoral Response and Cellular Response After Two Vaccine Doses

Pairwise comparisons were performed for each vaccine dose using the Wilcoxon test. Table 3 summarizes the changes observed after the second dose. Anti-SARS-CoV-2 IgG levels (BAU/mL) exhibited a significant increase (300-fold; *p* < 0.001), indicating a robust humoral response. Regarding the cell-mediated response, no significant changes were observed in CD3^+^, CD3^+^CD4^+^, CD3^+^CD8^+^, CD3^+^CD56^+^ (NKT), CD3^−^CD56^+^ (NK), or CD19^+^ T cells. Similarly, CD3^+^CD4^+^ T cell subpopulations (naïve, central memory, peripheral memory, and terminal effector memory T cells, TEMRA) remained unchanged. However, within the CD3^+^CD8^+^ T ell subpopulations, a significant increase was observed in naïve CD3^+^CD8^+^ T cells (1.36-fold; *p* = 0.05), while peripheral memory CD3^+^CD8^+^ T cells exhibited a statistically significant decrease (1.4-fold; *p* = 0.026). No significant differences were detected in other CD3^+^CD8^+^ subtypes.

In summary, the second vaccine dose elicited a strong humoral immune response, as evidenced by the significant increase in anti-SARS-CoV-2 IgG levels. While most cellular immunity parameters remained stable, the observed shifts in CD3^+^CD8^+^ T cell subsets suggest potential immune remodeling post-vaccination.

### 3.4. Effect of Booster Doses

Table 4 presents the results of pairwise comparisons performed using the Wilcoxon test to assess differences in humoral and cellular immunity parameters between the samples collected after the second vaccine dose and after the booster dose. 

Anti-SARS-CoV-2 IgG levels (BAU/mL) increased six-fold following the booster dose (*p* = 0.001), indicating a substantial improvement in the humoral response.

Cellular immunity showed that CD3^+^CD4^+^ T cells showed a moderate but significant 1.2-fold decrease (*p* = 0.031), while CD3^+^CD8^+^ T cells remained relatively stable, with no significant difference. CD3^+^CD56^+^ (NKT) cells exhibited a significant 6.9-fold decrease (*p* = 0.001), suggesting a pronounced impact on the natural killer T cell population. CD3−CD56^+^ (NK) cells and CD19^+^ (B cells) did not show statistically significant changes.

In the CD3^+^CD4^+^ T cell subsets, naïve CD3^+^CD4^+^ T cells decreased significantly post-booster, by 1.6-fold (*p* = 0.006). In contrast, central memory CD3^+^CD4^+^ T cells and peripheral memory CD3^+^CD4^+^ T cells significantly increased by 1.26-fold (*p* = 0.001) and 1.1-fold (*p* = 0.001), respectively.

In addition, in the CD3^+^CD8^+^ T cell subsets, naïve CD3^+^CD8^+^ T cells showed a significant 1.8-fold reduction (*p* = 0.001). Conversely, central memory CD3^+^CD8^+^ T cells and peripheral memory CD3^+^CD8^+^ T cells significantly increased by 1.64-fold (*p* = 0.001) and 1.9-fold (*p* = 0.001), respectively. CD3^+^CD8^+^ TEMRA cells significantly increased by 1.52-fold post-booster (*p* = 0.001), suggesting enhanced cytotoxic memory responses.

The booster dose elicited a stronger humoral immune response, as evidenced by the significant rise in anti-SARS-CoV-2 IgG levels. However, distinct changes in cellular immunity were observed, particularly a shift in CD3^+^CD4^+^ and CD3^+^CD8^+^ T cell memory populations. The concurrent increase in memory T cell subsets and reduction in naïve T cells suggests a potential transition towards a more antigen-experienced immune profile post-booster vaccination.

### 3.5. Antigen-Specific CD8^+^ T Cell Response

**Figure 3:**
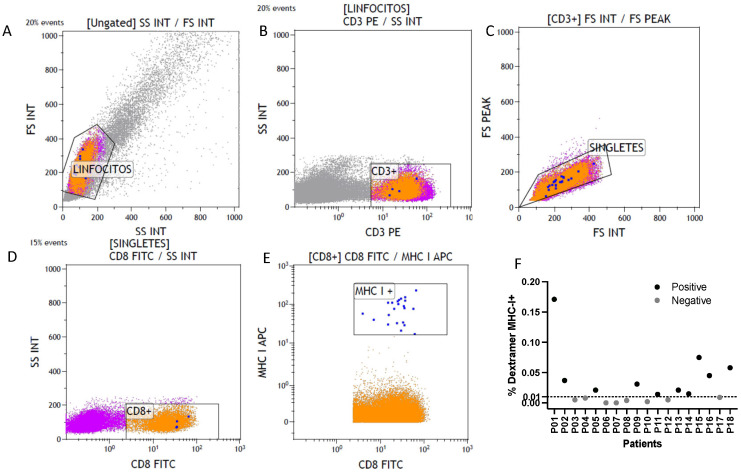
Flow cytometry gating strategy and detection of dextramer+ CD8^+^ T cells in breast cancer patients. (**A**) Forward scatter (FS) versus side scatter (SS) plot to gate lymphocytes; (**B**) Selection of CD3^+^ T cells within the lymphocyte gate; (**C**) Exclusion of doublets by FS area versus FS height; (**D**) Identification of CD8^+^ T cells within the CD3^+^ population; (**E**) Detection of MHC-I dextramer+ cells among CD8^+^ T cells. Representative dot plots are shown; positive events are indicated in blue; (**F**) Frequency of dextramer+ MHC-I^+^ CD8^+^ T cells in peripheral blood from individual patients. Each dot represents one patient; black = positive; gray = negative.

To assess SARS-CoV-2 specific CD8^+^ T cell responses, we used APC-labeled MHC I Dextramer reagents (Immudex) carrying the HLA-A*0201-restricted spike epitope YLQPRTFLL. The analysis was performed in blood samples from 18 patients collected after the administration of the booster dose. Among these, 10 patients (55.5%) developed detectable antigen-specific CD8^+^ T cells, while the remaining 8 patients (44.5%) did not show positive levels of SARS-CoV-2 specific cytotoxic T cells (Figure 3).

Clinical follow-up was conducted for one year after the booster dose. Only one patient (4.3%) developed SARS-CoV-2 infection, confirmed via PCR, 15 days after vaccination. The patient experienced mild upper respiratory symptoms that did not require hospitalization. Interestingly, this individual did not develop antigen-specific CD8^+^ T cells (MHC I dextramer < 0.01) but showed elevated levels of anti-SARS-CoV-2 IgG (8672.7 BAU/mL), which may explain the mild clinical course of the disease.

These findings indicate that the SARS-CoV-2 booster dose induces an antigen-specific cytotoxic T cell response in more than half of the patients. The absence of this response appears to be associated with increased susceptibility to viral infection. Moreover, our results suggest that antigen-specific CD8^+^ T cell responses and antibody levels may act as complementary but not directly correlated arms of immunity.

### 3.6. Relationship Between Antibody Levels and Cellular Immune Response

The correlation between antibody levels (Anti-SARS-CoV-2 IgG, BAU/mL) and various cellular immune parameters after the second dose of vaccine and the booster dose was assessed using Spearman’s test (Supplementary Table S2A,B).

**Figure 4:**
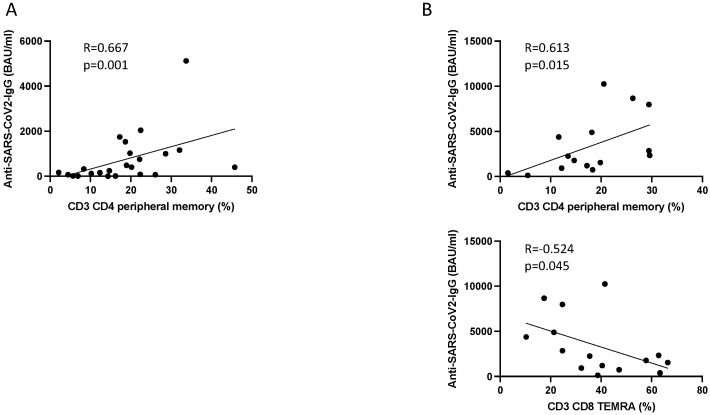
Correlation between antibody levels (BAU/mL) and the cellular immune response (%) assessed using the Spearman test. (**A**) After the second dose; (**B**) after the booster dose.

After the second dose, most correlations between Anti-SARS-CoV-2 IgG levels and cellular immune subsets were not statistically significant. Peripheral CD3^+^CD4^+^ memory T cells (%) showed a significant positive correlation with antibody levels (rho = 0.667, *p* = 0.001), suggesting a possible relationship between increased antibody production and expansion of memory CD4^+^ T cells (Figure 4A). 

After the booster dose, peripheral memory CD3^+^CD4^+^ T cells maintained a significant positive correlation with Anti-SARS-CoV-2 IgG levels (rho = 0.613, *p* = 0.015), reinforcing the association between booster-induced antibody responses and memory CD4^+^ T cells (Figure 4B). Furthermore, CD3^+^CD8^+^ TEMRA cells showed a significant negative correlation with IgG levels (rho = −0.524, *p* = 0.045), suggesting a possible inverse relationship between cytotoxic memory differentiation and humoral responses. While CD3^+^ T cells and CD3^+^CD4^+^ T cells showed moderately strong negative correlations with antibody level (rho = −0.509, *p* = 0.052 and rho = −0.463, *p* = 0.082, respectively), these did not reach statistical significance. 

Overall, these findings highlight the role of CD3^+^CD4^+^ peripheral memory T cells in antibody production, particularly following booster vaccination. The observed negative correlation between CD3^+^CD8^+^ TEMRA cells and IgG levels may suggest distinct pathways in cellular versus humoral immunity post-booster. Further investigations are warranted to elucidate the functional implications of these relationships.

Finally, the integration of data from the microbiota analysis with humoral assays using the diablo approach did not provide a clear differentiation when humoral and cellular immunity markers were used as diversity drivers.

### 3.7. Effect of Treatment Modality on Vaccine-Induced Immunity (Exploratory)

At baseline (*n* = 23; targeted therapy *n* = 11, chemotherapy *n* = 12), the targeted therapy group showed a higher percentage of CD19^+^ B cells and a higher fraction of naïve CD4^+^ T cells compared with chemotherapy (U = 9.0; *p* = 0.036 and U = 7.0; *p* = 0.020, respectively), with no other significant differences (Supplementary Table S3A). After the second dose, the only significant between-group difference was a lower proportion of CD3^+^CD56^+^ (NKT) cells in the targeted group (U = 29.0; *p* = 0.026); non-significant trends included higher anti–SARS-CoV-2 IgG with targeted therapy (*p* = 0.082) and higher CD8^+^ T-cell proportions with chemotherapy (*p* = 0.063) (Table S3B). After the booster, the targeted group showed lower CD4^+^ central memory (U = 19.0; *p* = 0.027), whereas IgG titers and MHC-I dextramer positivity (spike-specific CD8^+^) were comparable between groups (*p* = 0.327 and *p* = 0.540) (Table S3C). These results indicate modest, treatment-dependent immunomodulation—primarily affecting NKT cells and CD4^+^ central memory—while humoral responses appear broadly similar across modalities. In general, eventual differences among treatments, considering the doses at once, in terms of alpha diversity indexes, did not reveal any microbiome structure population shift (Supplementary Figure S1). Given the small sample size and multiple comparisons, these findings are exploratory and hypothesis-generating. 

## 4. Discussion

In this prospective observational study, we investigated the immunogenicity of the mRNA vaccine against SARS-CoV-2 in a cohort of 23 breast cancer patients undergoing active cancer treatment. Our results reveal that, despite the immunosuppressive context, these patients were able to mount detectable humoral and cellular immune responses after two doses and a booster, with several immunological patterns emerging in relation to the type of treatment and the composition of the gut microbiota.

In line with other studies in cancer and immunocompromised populations [1,2,4,31], we observed a pronounced increase in anti-SARS-CoV-2 IgG antibody titers after the second dose of the vaccine (~300-fold) and an even stronger response after the booster dose (~2200-fold). This demonstrates that breast cancer patients retained the ability to elicit a strong antibody-mediated response, despite ongoing cytotoxic or targeted therapies. This response was accompanied by a simultaneous increase in circulating B cells (CD19^+^), further supporting the idea that B cell activation plays a key role in humoral immunity after vaccination. Studies such as those by Wan et al. have linked elevated antibody levels with increased susceptibility to SARS-CoV-2 infection through a mechanism that mediates the entry of the virus into the cell through the Fc region of immunoglobulins. In contrast, the follow-up performed in our study only showed one patient to be infected after the booster dose [32].

In addition to the global immunophenotyping, we further characterized antigen-specific cytotoxic T cell immunity using MHC I dextramer technology. More than half of the patients (55.5%) developed detectable spike-specific CD8^+^ T cells, while the remaining 44.5% failed to mount this response. Interestingly, the only patient who developed PCR-confirmed SARS-CoV-2 infection during follow-up lacked dextramer-positive CD8^+^ T cells but showed high IgG titers. This observation reinforces the notion that antibodies may be sufficient to prevent severe outcomes but not necessarily infection, while CD8^+^ T-cell responses contribute to limiting susceptibility. Similar findings have been reported in other immunocompromised cohorts, where robust CD8^+^ responses were associated with protection from breakthrough infections [33,34]. Together, our data suggest that humoral immunity and cytotoxic T cell immunity act as complementary but not redundant arms of protection and that monitoring both parameters may be particularly relevant in cancer population.

On the other hand, cell-mediated immune responses showed more nuanced dynamics. Although the total percentages of CD3^+^, CD4^+^, and CD8^+^ T cells remained relatively stable, immunophenotyping showed significant shifts toward memory phenotypes after the booster dose. Specifically, we observed a reduction in naïve CD4^+^ and CD8^+^ T cells with an increase in core memory (CM) and effector memory (EM) subsets, indicating effective activation and maturation of antigen-specific T cells [33,34,35]. These changes are essential markers of long-lasting cellular immunity and are consistent with the memory expansions described in other vaccinated cohorts [36,37,38].

It is noteworthy that NKT cells showed a transient increase after the second dose, followed by a sharp decline after the booster, suggesting an early interaction between the innate and adaptive immune systems that is subsequently resolved. The increase in CD8^+^ TEMRA cells—terminally differentiated effector memory cells—after the booster dose points to the generation of cytotoxic memory T cells with potential antiviral capacity (Table 4) [39,40]. However, these cells showed a negative correlation with IgG levels, highlighting a possible immunological divergence between humoral and cytotoxic responses. This phenomenon has also been observed in studies of chronic viral infections and cancer immunotherapy, where increased cytotoxicity may be accompanied by weaker antibody production [41,42]. This is due to CD8^+^ TEMRA cells, which are associated with aging and immune exhaustion (immunosenescence) as a consequence of exposure to a chronic inflammatory environment, and this may affect the production of high-affinity IgGs [43,44].

One of the most notable findings was the strong positive correlation between peripheral memory CD4^+^ T cells and IgG levels after the second dose (ρ = 0.667, *p* = 0.001) and booster (ρ = 0.613, *p* = 0.015). Peripheral memory CD4^+^ cells are known to provide rapid and effective helper functions to B cells, including cytokine secretion (e.g., IL-4, IL-5, IL-21), which facilitates class switch recombination and plasma cell differentiation. This relationship highlights the critical role of CD4^+^ memory T cells in maintaining and amplifying humoral responses over time.

Regarding the composition of the microbiota, our exploratory analysis revealed suggestive trends rather than statistically conclusive results, given the small sample size. However, multivariate methods (sPLS-DA, mixOmics) identified bacterial genera such as *Alistipes*, *Romoutsia*, and *Butyricimonas* as enriched in patients with high response. These taxa are known producers of short-chain fatty acids (SCFAs) and have previously been linked to increased vaccine efficacy through dendritic cell maturation and follicular helper T cell (Tfh) support [11,45,46]. In contrast, genera such as *Weissella* and *Slackia* were associated with a lower response, suggesting possible immunomodulatory or inhibitory effects. Although alpha diversity did not differ significantly between treatment groups or responders, these changes in composition provide a basis for future research (Table 5).

While our study is limited by its sample size and treatment heterogeneity, its strengths lie in its prospective longitudinal design and comprehensive immunological profile. The simultaneous monitoring of antibody titers, T cell phenotypes, and microbiota signatures at three key time points provides an integrated perspective on the dynamics of the vaccine response in this vulnerable population. Importantly, the mRNA vaccine was well tolerated, with no serious adverse events or hospitalizations reported. Finally, it is important to consider patient adherence to booster recommendations. Our updated follow-up until 2024 shows that only eight of the original study participants continue receiving annual booster doses, while the majority of patients declined further vaccination. This highlights the challenge of vaccine acceptance in this vulnerable population, despite evidence of robust immunogenicity and the potential protective role of combined humoral and cytotoxic responses.

Therefore, breast cancer patients undergoing active treatment can generate robust immune responses to SARS-CoV-2 vaccination, especially after the booster dose. These responses are characterized by high IgG levels, expansion of memory T cell subsets, and associations with potentially favorable gut microbiota profiles. The positive relationship observed between memory CD4^+^ T cells and antibody production highlights the importance of immune memory in long-term protection. Furthermore, our findings support the idea that gut microbial composition may modulate vaccine efficacy, underscoring the value of incorporating microbiota analysis into future vaccine optimization strategies for immunocompromised hosts.

## Figures and Tables

**Table 1:** Baseline clinical characteristics of study population.

Variable	*N* = 23
Age (median, IQR)	54 (45–76)
Breast tumor phenotype	
Luminal HER-2 Triple-negative	11 (48%) 9 (39%) 3 (13%)
Hormonal status	
Pre-menopausal Post-menopausal	11 (48%) 12 (52%)
Presence of comorbidity	
Hypertension Allergy Dyslipidemia Hypothyroidism Diabetes Other	1 (4%) 1 (4%) 2 (8%) 2 (8%) 1 (4%) 2 (8%)
Cancer status	
Remission or no evidence of disease Present, stable Present and metastasis	7 (30%) 11 (48%) 5 (22%)
Current medication	
Target therapy Chemotherapy	11 (48%) 12 (52%)
Outcomes	
Hospitalization Intensive care unit Death	0 (0%) 0 (0%) 1 (4%)
Smoking habits	
Ex-smoker Smoker Non-smoker	0 (0%) 3 (13%) 20 (87%)

**Table 2:** Results of the Friedman test studying the relationships between IgG Anti-SARS-CoV-2 antibody levels (BAU/mL) and the cellular immune response.

	Baseline Median	2nd Dose Median	Booster Dose Median	Friedman	*p*
Anti-SARS-CoV-2 IgG (BAU/mL)	0.000	327.026	2253.770	21.535	0.000
CD3^+^ T cells (%)	19.030	18.200	17.460	16.545	0.120
CD3^+^CD4^+^ T cells (%)	33.595	45.610	37.710	3.818	0.148
CD3^+^CD8^+^T Cells (%)	21.305	21.460	25.270	2.364	0.307
CD3^+^CD56^+^ (NKT, %)	4.455	6.800	0.980	17.636	0.000
CD3^-^CD56^+^ (NK, %)	16.100	15.690	11.560	3.818	0.148
CD19^+^ T cells (%)	3.975	8.390	11.430	6.837	0.033
CD3^+^CD4^+^ T cells naïve (%)	20.780	28.350	17.590	7.818	0.020
CD3^+^CD4^+^ T cells central memory (%)	45.760	49.790	63.240	7.818	0.020
CD3^+^CD4^+^ peripheral memory (%)	27.375	17.250	18.140	2.364	0.307
CD3^+^CD4^+^ TEMRA* (%)	1.455	1.260	0.870	1.273	0.529
CD3^+^CD8^+^ naïve (%)	29.580	40.370	22.260	6.545	0.038
CD3^+^CD8^+^ central memory (%)	7.825	7.840	12.860	5.091	0.078
CD3^+^CD8^+^ peripheral memory (%)	15.850	11.480	21.860	15.273	0.000
CD3^+^CD8^+^ TEMRA* (%)	27.175	25.510	38.710	1.273	0.529

TEMRA* = terminal effector memory T cells.

**Table 3:** Pairwise comparisons (Wilcoxon test) of the determined parameters of humoral and cellular immunity between samples obtained at the baseline (B) and second-dose time points (2).

Doses		Median	Wilcoxon Z	*p*-value
Anti-SARS-CoV2 IgG (BAU/mL)	B	0.000	−4.015	0.000
	2	327.026		
CD3^+^ T cells (%)	B	19.030	−1.344	0.179
	2	18.200		
CD3^+^CD4^+^ T cells (%)	B	33.595	−1.293	0.196
	2	45.610		
CD3^+^CD8^+^T Cells (%)	B	21.305	−1.138	0.255
	2	21.460		
CD3^+^CD56^+^ (NKT, %)	B	4.455	−1.189	0.234
	2	6.680		
CD3^+^CD56^+^ (NK, %)	B	16.100	−0.103	0.918
	2	15.690		
CD19^+^ T cells (%)	B	3.975	−0.982	0.326
	2	8.390		
CD3vCD4^+^ T cells naïve (%)	B	20.780	−0.621	0.535
	2	28.350		
CD3^+^CD4^+^ T cells central memory (%)	B	45.760	−1.500	0.134
	2	49.790		
CD3^+^CD4^+^ peripheral memory (%)	B	27.375	−1.500	0.134
	2	17.250		
CD3^+^CD4^+^ TEMRA* (%)	B	1.455	−0.414	0.679
	2	1.260		
CD3^+^CD8^+^ naïve (%)	B	29.580	−1.913	0.050
	2	40.370		
CD3^+^CD8^+^ central memory (%)	B	7.825	−0.621	0.535
	2	7.840		
CD3^+^CD8^+^ peripheral memory (%)	B	15.850	−2.223	0.026
	2	11.480		
CD3^+^CD8^+^ TEMRA* (%)	B	27.175	−1.551	0.121
	2	25.510		

TEMRA* = terminal effector memory T cells.

**Table 4:** Pairwise comparisons (Wilcoxon test) of the determined parameters of humoral and cellular immunity between the samples obtained at the time points after the second dose (2) and the booster dose (Booster).

	Doses	Median	Wilcoxon Z	*p*-Value
Anti-SARS-CoV2 IgG (BAU/mL)	2	327.026	−3.237	0.001
	Booster	2253.770		
CD3^+^ T cells (%)	2	18.200	−1.343	0.179
	Booster	17.460		
CD3^+^CD4^+^ T cells (%)	2	45.610	−2.158	0.031
	Booster	37.710		
CD3^+^CD8^+^T Cells (%)	2	21.460	−0.114	0.910
	Booster	25.270		
CD3^+^CD56^+^ (NKT, %)	2	6.800	−3.408	0.001
	Booster	0.980		
CD3^-^CD56^+^ (NK, %)	2	15.690	−1.363	0.173
	Booster	11.560		
CD19^+^ T cells (%)	2	8.390	−1.915	0.056
	Booster	11.430		
CD3^+^CD4^+^ T cells naïve (%)	2	28.350	−2.727	0.006
	Booster	17.590		
CD3^+^CD4^+^ T cells central memory (%)	2	49.790	−3.408	0.001
	Booster	63.240		
CD3^+^CD4^+^ peripheral memory (%)	2	17.250	−3.237	0.001
	Booster	18.140		
CD3^+^CD4^+^ TEMRA* (%)	2	1.260	−1.136	0.256
	Booster	0.870		
CD3^+^CD8^+^ naïve (%)	2	40.370	−3.237	0.001
	Booster	22.260		
CD3^+^CD8^+^ central memory (%)	2	7.840	−3.237	0.001
	Booster	12.860		
CD3^+^CD8^+^ peripheral memory (%)	2	11.480	−3.237	0.001
	Booster	21.860		
CD3^+^CD8^+^ TEMRA* (%)	2	25.510	−3.237	0.001
	Booster	38.710		

TEMRA* = terminal effector memory T cells.

**Table 5:** Immunological characteristics of selected gut bacteria associated with vaccine response in cancer patients.

Bacterial Genus	Functional Type	Immunological Role	Potential Impact on Vaccine Response	References
*Alistipes*	SCFA producer (propionate, butyrate)	Modulates inflammation; promotes regulatory T cell and Th1 differentiation	Positive—associated with enhanced immune response	[11,15,46,47,48]
*routyRomboutsia*	SCFA producer (acetate)	Associated with gut homeostasis and symbiosis; potential immune modulation	Possibly positive—requires further study	[15,49]
*Butyricimonas*	SCFA producer (butyrate)	Enhances dendritic cell maturation and Tfh cell differentiation; supports IgG production	Positive—supports humoral response	[11,15,50]
*Weissella*	Lactic acid bacterium (facultative)	Reported as a pathobiont in immunocompromised hosts; linked to dysbiosis and inflammation	Negative—potentially inhibits vaccine efficacy	[51]
*Slackia*	Actinobacterium, metabolizes isoflavones	Associated with dysbiosis, inflammation, and metabolic disruption	Negative—linked to poor immune modulation	[52,53]

SCFAs: Short-chain fatty acids (e.g., butyrate, propionate, acetate), known to support anti-inflammatory immune pathways; Tfh cells: T follicular helper cells, critical for B-cell activation and high-affinity antibody production.

## Data Availability

The raw sequencing data obtained during the current study are available in the SRA repository with the accession number PRJEB94018. [https://www.ncbi.nlm.nih.gov/bioproject/PRJEB94018 (accessed on 25 July 2025)].

## Supplementary Materials

The following supporting information can be downloaded at: https://www.mdpi.com/article/10.3390/pathogens14090947/s1. The supplementary material includes the following: Figure S1: Boxplots of main alpha diversity indexes. Y-axes report the index. x-axis reports the comparison among groups. Upper comparisons between boxplots indicate the *p*-value of the Wilcoxon test between the groups. Box a, shows the differences between the therapy groups. Box b shows differences between the antibodies responses. Dots are colored according to the vaccination dose. Table S1: Post-vaccination symptoms. Table S2A: Determination of the relationship between antibody levels (Anti-SARS-CoV-2 IgG (BAU/mL) produced and the cellular immune response after the second dose using the Spearman test. Table S2B: Spearman test: Anti-SARS-CoV-2 IgG (BAU/mL) vs. booster doses. Table S3A: Baseline immune parameters by treatment modality (targeted therapy vs chemotherapy). Table S3B: Post-second-dose immune parameters by treatment modality. Table S3C: Post-booster immune parameters and antigen-specific CD8^+^ T cell positivity by treatment modality.
