# Supplementary material for: Immunogenicity of SARS-CoV-2 mRNA Vaccine in Breast Cancer Patients Undergoing Active Treatment: A Prospective Observational Study
Source: Pathogens. 2025 Sep 18;14(9):947. doi: 10.3390/pathogens14090947 (PMC12472335; doi:10.3390/pathogens14090947)
Supplement: Supplementary file 1 [file pathogens-14-00947-s001.zip › Supplementary_material_rev2.pdf]

Supplementary material

Table S1: Post-vaccination symptoms

	2 doses	Blooster Doses
	%	%
Cough	4.3	0
Fever	21.7	20
Dyspnea	8.7	0
Odynophagia	13	6.7
Vomits	0	0
Diarrhea	0	0
Asthenia	47.8	26.7
Anosmia	17.4	0
Bodyache	47.8	26.7
Headache	34.8	20
Redness_site	13	0
Pain site	87	92.9
Other	4.3	0
Reduction of activity	26.1	20
Medical consultation	4.3	0
Medication*	34.8	33.3
Hospitalization	0	0

* antipyretic

Determination of the relationship between antibody levels (Anti-SARS-CoV-2 IgG (BAU/mL) produced and the cellular immune response using ***the Spearman test.***

Table S2 A: Spearman test - Anti-SARS-CoV-2 IgG (BAU/mL) vs 2ª doses

	**Rho de Spearman**	**p**
CD3+ T cells (%)	-0.101	0.647
CD3+CD4+ T cells (%)	-0.185	0.399
CD3+ CD8+T Cells (%)	0.095	0.667
CD3+CD56+ (NKT, %)	-0.015	0.946
CD3-CD56+(NK, %)	-0.016	0.943
CD19 + T cells (%)	0.303	0.159
CD3+CD4 + T cells naive (%)	-0.295	0.171
CD3+CD4+ T cells central memory (%)	-0.047	0.830
CD3+CD4+ peripheral memory (%)	0.667	0.001
CD3+CD4+ *TEMRA* (%)*	0.204	0.352
CD3+CD8+ *naive (%)*	-0.207	0.344
CD3+CD8+ central memory (%)	-0.217	0.319
CD3+CD8+ peripheral memory (%)	0.110	0.618
CD3+CD8+TEMRA*(%)	0.058	0.792

Table S2 B: Spearman test - Anti-SARS-CoV-2 IgG (BAU/mL) vs Booster doses

	**Rho de Spearman**	**p**
CD3+ T cells (%)	-0.509	0.052
CD3+CD4+ T cells (%)	-0.463	0.082
CD3+ CD8+T Cells (%)	0.102	0.718
CD3+CD56+ (NKT, %)	-0.125	0.657
CD3-CD56+(NK, %)	0.318	0.248
CD19 + T cells (%)	0.032	0.909
CD3+CD4 + T cells naive (%)	0.027	0.924
CD3+CD4+ T cells central memory (%)	-0.005	0.985
CD3+CD4+ peripheral memory (%)	0.613	0.015
CD3+CD4+ *TEMRA* (%)*	-0.191	0.495
CD3+CD8+ *naive (%)*	0.141	0.616
CD3+CD8+ central memory (%)	-0.020	0.945
CD3+CD8+ peripheral memory (%)	0.427	0.112
CD3+CD8+TEMRA*(%)	-0.524	0.045

Effect of treatment modality on vaccine-induced immunity (exploratory)

Table S3A: Baseline immune parameters by treatment modality (targeted therapy vs chemotherapy).

	Therapy	Mean rank	U	p
CD3+ T cells (%)	Targeted therapy	8.00	25.000	0.777
	Chemotherapy	8.73		
CD3+CD4+ T cells (%)	Targeted therapy	8.00	25.000	0.777
	Chemotherapy	8.73		
CD3+CD8+T Cells (%)	Targeted therapy	9.40	23.000	0.610
	Chemotherapy	8.09		
CD3+CD56+ (NKT, %)	Targeted therapy	7.20	21.000	0.462
	Chemotherapy	9.09		
CD3-CD56+ (NK, %)	Targeted therapy	9.40	23.000	0.610
	Chemotherapy	8.09		
CD19 + T cells (%)	Targeted therapy	11.00	9.000	0.036
	Chemotherapy	7.36		
CD3+CD4 + T cells naive (%)	Targeted therapy	8.80	7.000	0.020
	Chemotherapy	8.36		
CD3+CD4+ T cells central memory (%)	Targeted therapy	9.00	22.000	0.533
	Chemotherapy	8.27		
CD3+CD4+ peripheral memory (%)	Targeted therapy	8.80	25.000	0.777
	Chemotherapy	8.36		
CD3+CD4+ *TEMRA* (%)*	Targeted therapy	8.60	27.000	0.955
	Chemotherapy	8.36		
CD3+CD8+ *naive (%)*	Targeted therapy	7.80	13.000	0.100
	Chemotherapy	8.82		
CD3+CD8+ central memory (%)	Targeted therapy	9.50	22.500	0.571
	Chemotherapy	8.05		
CD3+CD8+ peripheral memory (%)	Targeted therapy	10.00	20.000	0.396
	Chemotherapy	7.82		
CD3+CD8+ *TEMRA* (%)*	Targeted therapy	8.40	27.000	0.955
	Chemotherapy	8.55		

For each outcome, the Mann–Whitney **mean rank** is shown for each group; a higher mean rank indicates larger values in that group. **U** denotes the Mann–Whitney statistic and **p** the two-sided nominal p-value (p<0.05 considered statistically significant).

Abbreviations: BAU/mL, binding antibody units per milliliter; **NKT**, $CD3^+CD56^+$ natural killer T cells; **NK**, $CD3^-CD56^+$ natural killer cells; **$CD19^+$**, B cells; **TEMRA**, terminally differentiated effector memory RA^+ T cells.

Table S3B: Post-second-dose immune parameters by treatment modality.

	Therapy	Mean rank	U	p
Anti-SARS-CoV2-IgG (BAU/mL)	Targeted therapy	14.80	37.000	0.082
	Chemotherapy	9.85		
CD3+ T cells (%)	Targeted therapy	11.40	59.000	0.710
	Chemotherapy	12.46		
CD3+CD4+ T cells (%)	Targeted therapy	12.60	59.000	0.710
	Chemotherapy	11.54		
CD3+CD8+T Cells (%)	Targeted therapy	9.00	35.000	0.063
	Chemotherapy	14.31		
CD3+CD56+ (NKT, %)	Targeted therapy	8.40	29.000	0.026
	Chemotherapy	14.77		
CD3-CD56+(NK, %)	Targeted therapy	11.50	60.000	0.756
	Chemotherapy	12.38		
CD19 + T cells (%)	Targeted therapy	14.40	41.000	0.137
	Chemotherapy	10.15		
CD3+CD4 + T cells naive (%)	Targeted therapy	13.80	47.000	0.264
	Chemotherapy	10.62		
CD3+CD4+ T cells central memory (%)	Targeted therapy	9.60	41.000	0.137
	Chemotherapy	13.85		
CD3+CD4+ peripheral memory (%)	Targeted therapy	12.30	62.000	0.852
	Chemotherapy	11.77		
CD3+CD4+ *TEMRA* (%)*	Targeted therapy	12.90	56.000	0.577
	Chemotherapy	11.31		
CD3+CD8+ *naive (%)*	Targeted therapy	9.80	46.000	0.239
	Chemotherapy	13.69		
CD3+CD8+ central memory (%)	Targeted therapy	12.40	64.000	0.951
	Chemotherapy	11.69		
CD3+CD8+ peripheral memory (%)	Targeted therapy	13.90	46.000	0.239
	Chemotherapy	10.54		
CD3+CD8+ *TEMRA* (%)*	Targeted therapy	12.10	64.000	0.951
	Chemotherapy	11.92		

For each outcome, the Mann–Whitney **mean rank** is shown for each group; a higher mean rank indicates larger values in that group. **U** denotes the Mann–Whitney statistic and **p** the two-sided nominal p-value (p<0.05 considered statistically significant).

Abbreviations: BAU/mL, binding antibody units per milliliter; **NKT**, $CD3^{+}CD56^{+}$ natural killer T cells; **NK**, $CD3^{-}CD56^{+}$ natural killer cells; **$CD19^{+}$**, B cells; **TEMRA**, terminally differentiated effector memory RA^{+} T cells.

Table S3C: Post-booster immune parameters and antigen-specific CD8⁺ T-cell positivity by treatment modality.

	Therapy	Mean rank	U	p
Anti-SARS-CoV2-IgG (BAU/mL)	Targeted therapy	6.40	17.000	0.327
	Chemotherapy	8.80		
Dextramer+ MHC-I+ CD8+ T	Targeted therapy	7.00	20.000	0.540
	Chemotherapy	8.50		
CD3+ T cells (%)	Targeted therapy	7.40		
	Chemotherapy	10.20	14.000	0.513
CD3+CD4+ T cells (%)	Targeted therapy	6.90		
	Chemotherapy	10.60	12.000	0.111
CD3+CD8+T Cells (%)	Targeted therapy	6.70		
	Chemotherapy	5.80	23.000	0.806
CD3+CD56+ (NKT, %)	Targeted therapy	9.10		
	Chemotherapy	7.60	23.000	0.245
CD3-CD56+(NK, %)	Targeted therapy	8.20		
	Chemotherapy	7.60	21.000	0.624
CD19 + T cells (%)	Targeted therapy	8.20		
	Chemotherapy	8.40	20.000	0.540
CD3+ T cells (%)	Targeted therapy	7.80		
CD3+CD4 + T cells naive (%)	Targeted therapy	10.40	17.000	0.327
	Chemotherapy	6.80		
CD3+CD4+ T cells central memory (%)	Targeted therapy	6.80	19.000	0.027
	Chemotherapy	8.60		
CD3+CD4+ peripheral memory (%)	Targeted therapy	4.40	19.000	0.462
	Chemotherapy	9.80		
CD3+CD4+ *TEMRA* (%)*	Targeted therapy	6.60	22.000	0.513
	Chemotherapy	8.70		
CD3+CD8+ *naive (%)*	Targeted therapy	7.60	16.000	0.710
	Chemotherapy	8.20		
CD3+CD8+ central memory (%)	Targeted therapy	9.20	19.000	0.462
	Chemotherapy	7.40		
CD3+CD8+ peripheral memory (%)	Targeted therapy	8.80	21.000	0.624
	Chemotherapy	7.60		
CD3+CD8+ *TEMRA* (%)*	Targeted therapy	6.20	16.000	0.270
	Chemotherapy	8.90		

For each outcome, the Mann–Whitney **mean rank** is shown for each group; a higher mean rank indicates larger values in that group. **U** denotes the Mann–Whitney statistic and **p** the two-sided nominal p-value ($p<0.05$ considered statistically significant).

Abbreviations: BAU/mL, binding antibody units per milliliter; **NKT**, $CD3^+CD56^+$ natural killer T cells; **NK**, $CD3^-CD56^+$ natural killer cells; **$CD19^+$**, B cells; **TEMRA**, terminally differentiated effector memory RA^+ T cells.
