# Supplementary figures and images for: Immunogenicity of SARS-CoV-2 mRNA Vaccine in Breast Cancer Patients Undergoing Active Treatment: A Prospective Observational Study
Source: Pathogens. 2025 Sep 18;14(9):947. doi: 10.3390/pathogens14090947 (PMC12472335; doi:10.3390/pathogens14090947)

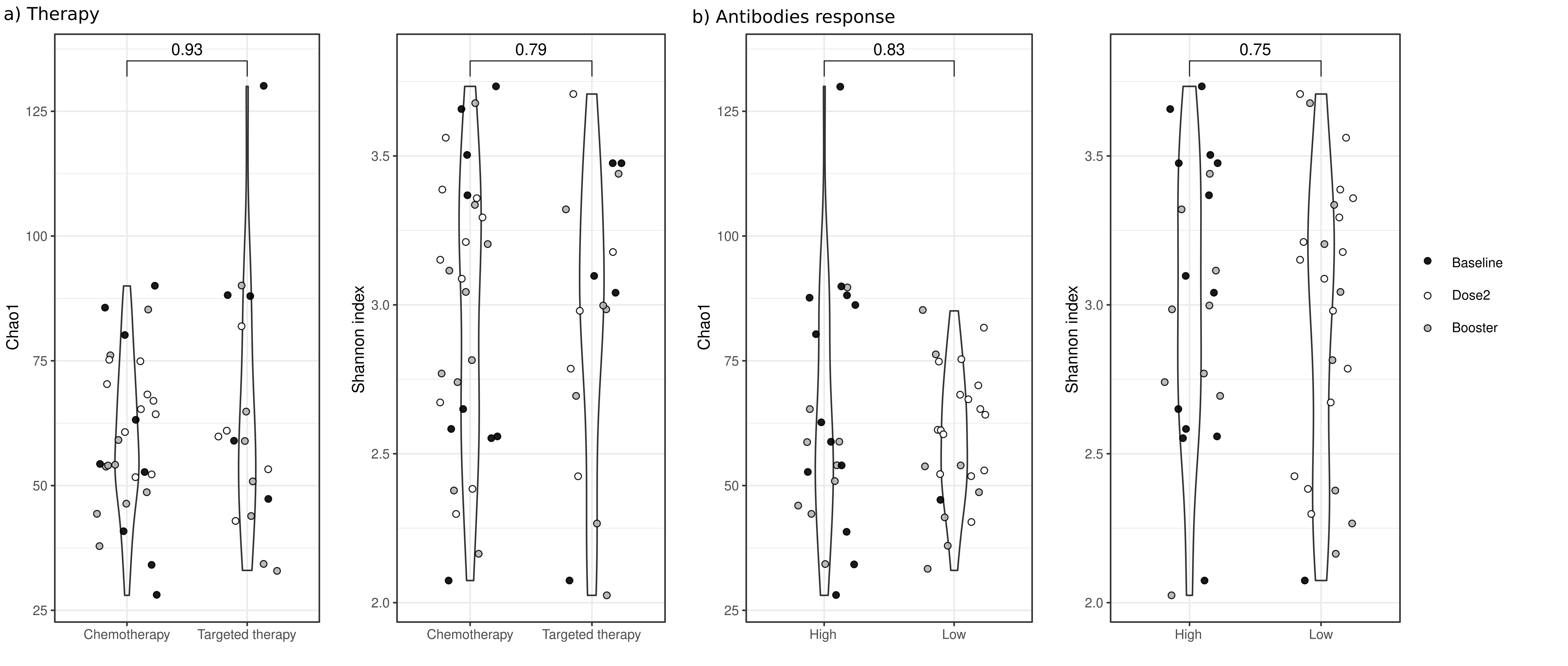

Supplement: Supplementary file 1 [file pathogens-14-00947-s001.zip › Suppl1_bw_v2.tiff]
